# Palliative expeditiously adaptive quad shot radiotherapy for head and neck cancers (PEAQ-RT)

**DOI:** 10.1016/j.ctro.2025.101012

**Published:** 2025-07-08

**Authors:** Weiren Liu, Joshua P. Schiff, Comron Hassanzadeh, Karen Miller, Casey Hatscher, Robbie Beckert, Alex Price, Mackenzie Daly, Randall Brenneman, Lauren Henke, Anthony Apicelli, Michael Moravan, Wade Thorstad, Eric Laugeman

**Affiliations:** a Department of Radiation Oncology, Washington University School of Medicine in St. Louis, St. Louis, MO, United States; b Department of Radiation Oncology, Keck School of Medicine of USC, Norris Comprehensive Cancer Center. Los Angeles, CA, United States; c Division of Radiation Oncology, MD Anderson Cancer Center, Houston, TX, United States; d Department of Radiation Oncology, University Hospitals, Case Western Reserve University,Cleveland, OH, United States; e Department of Radiation Oncology, University of Colorado School of Medicine, Colorado Springs, CO, United States; f Radiation Oncology, Springfield Clinic, Springfield, IL, United States

**Keywords:** Quad shot, Adaptive radiotherapy, Online adaptation, CT-guided adaptive radiotherapy, Treatment burden reduction, Patient centered care, Head and neck cancer, Advanced head and neck cancer

## Abstract

**Purpose/objective:**

Quad shot radiotherapy (QS-RT) is integral to head and neck cancer palliative care, but multiple CT-simulations for QS-RT cycles can be burdensome for patients. We evaluated the ability of an online adaptive radiotherapy (ART) workflow (PEAQ-RT) to eliminate extra CT simulations in QS-RT and reduce treatment related burdens.

**Materials/methods:**

Ten patients with head and neck malignancies were enrolled in this prospective study receiving QS-RT for up to three cycles, each comprising four fractions of 350 cGy delivered twice daily, with a total dose of 1400 cGy per cycle. QS-RT could be delivered up to three cycles, spaced three to four weeks apart. Patients underwent standard CT simulation, and the simulation plan served as the treatment plan for the first QS-RT cycle. For subsequent QS-RT cycles, patients proceeded directly to adaptive treatment via institutional online ART protocol. Feasibility was defined as completing this expedited adaptive QS-RT workflow in at least 80 % of attempted adapted fractions.

**Results:**

Ten patients aged 56–89 were enrolled. Eight patients received a second cycle of QS-RT and four patients received a third cycle. PEAQ-RT workflow was feasible in 87.5% (7/8) of patients who received at least one adapted cycle and was feasible in 86% (12/14) of attempted adapted fractions. For the second and third cycles, average total workflow time for the adaptive treatments was 28 min (14–38). All constraint violations were resolved with the use of online adaptation. The PEAQ-RT workflow eliminated a median of 2 (range: 0–2) simulation visits with additional QS-RT cycles. This resulted in a median travel distance savings of 50.8 miles (range: 40.6–848 miles) and a median reduction of 3.5 h in travel time per patient.

**Conclusion:**

PEAQ-RT enabled QS-RT while eliminating the need for additional CT simulation appointments for subsequent cycles.

## Highlights

• Quad shot is a safe and effective treatment for advanced head and neck cancer.
• Additional appointments between cycles can be onerous for this patient population.
• CT-guided online adaptive workflow (PEAQ-RT) eliminates extra simulation visits.
• PEAQ-RT is feasible and reduces both travel distance and time burdens for patients.

## Introduction

The Quad Shot radiotherapy (QS-RT) regimen has been demonstrated to relieve symptoms including pain, dysphagia, and bleeding in patients with incurable head and neck cancers [[1], [2], [3]]. The regimen involves prescribing 1400 cGy in four twice-daily fractions followed by additional cycles at 3–4 week intervals up to three cycles [1]. While effective, the cyclic nature of QS-RT means that patients will have scheduled appointments for response and toxicity assessment, repeat CT simulation, a break for re-planning, and then a return visit for additional treatment. These factors place a significant burden on patients, especially those in rural areas or with transportation challenges.

Online adaptive radiotherapy (ART) represents a potential avenue to increase the efficiency of QS-RT. ART is an image-guided radiotherapy treatment technique that involves re-contouring and re-optimizing a patient’s treatment plan based on their anatomy-of-the-day, all while the patient is on the treatment table. ART has been demonstrated to improve the dosimetry of radiotherapy including resolving organ at risk (OAR) constraints and improving target coverage, which has conferred improved disease outcome and toxicity benefits in a variety of disease sites [[4], [5], [6], [7], [8]]. By accounting for anatomic changes between cycles, ART may also offer workflow and efficiency advantages in the case of QS-RT by eliminating the need for re-simulation prior to additional treatment cycles. We hypothesized that an online ART-based QS-RT workflow would be feasible and could deliver QS-RT without requiring additional CT simulation appointments. This hypothesis and workflow was tested in this pilot study: palliative expeditiously adaptive quad shot radiotherapy (PEAQ-RT).

## Methods/materials

### Patient characteristics

This prospective clinical trial (NCT04379505) was approved by the Institutional Review Board. Eligible patients were ≥18 years old with primary or metastatic head and neck malignancies suitable for PEAQ-RT. Patients with prior head and neck radiotherapy were included on this study.

### Study intervention

PEAQ-RT radiation therapy was delivered over four, twice-daily fractions of 350 cGy per fraction at least 6 h apart over two days for a total dose of 1400 cGy per cycle. If applicable, cycles 2 and 3 were delivered in 3- to 4-week intervals for cumulative dose of 4200 cGy in 12 fractions. GTV is gross tumor volume as defined by treating physician. There is no CTV expansion and 5 mm expansion around GTV for PTV. Prescription dose was delivered to the PTV with dose adaptation, if necessary, based on safety constraints that are already approved to prevent OAR injury. OAR constraints were based on single cycle of PEAQ-RT. For radiation naïve patients, max dose to brain, spinal cord and brainstem was 1400 cGy while for patients with prior radiation history, max dose to brain, spinal cord and brain stem was 200 cGy, 170 cGy and 200 cGy respectively. Hotspots were avoided on mandible [9]. ART was delivered with CT-guidance (CTgART) on the ETHOS platform (Varian Medical Systems, Palo Alto, CA) [10].

### CT simulation and PEAQ-RT cycle 1

**Fig. 1:**
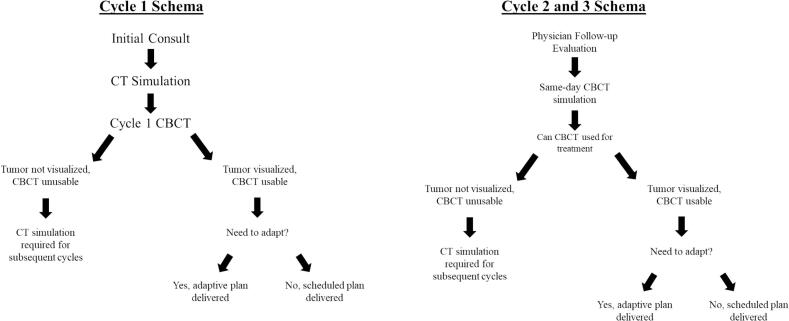
PEAQ-RT work flow for cycle 1 and cycle 2/3.

PEAQ-RT workflow is detailed in Fig. 1. For the first cycle of PEAQ-RT, patients proceeded with standard CT simulation with IV contrast for treatment planning. Treatment planning was performed in the ETHOS treatment planning system. At the time of fraction 1, an initial cone-beam CT (CBCT) was acquired and reviewed by the treating physician to determine if the CBCT quality was adequate for target and OAR delineation. The baseline simulation plan was used for all fractions of cycle 1.

### PEAQ-RT cycle 2 and cycle 3

At the discretion of the treating radiation oncologist, patients were followed up 3 to 4 weeks after cycle 1 to determine whether additional cycles of PEAQ-RT were needed. For patients proceeding to cycle 2 and cycle 3 of PEAQ-RT, patients underwent a CTgART session for the first fraction of cycle 2 and cycle 3. Prior to delivery of fraction 1 of cycle 2, a radiation oncologist reviewed CBCT images and determined if target and OAR were adequately delineated and if the existing treatment plan adequately covered the target volumes. A decision was then made to adapt or proceed with the simulation plan for fraction 1 of cycle 2 or 3. A CT simulation appointment slot was reserved in cases where GTV was not identifiable on CBCT or if the adapted and scheduled plans were deemed unacceptable by the physician. Only fraction 1 of cycle 2 and 3 was adapted. Given that fractions 2–4 of cycle 2 and 3 are delivered within 2 days, the delivered treatment plan from fraction 1 was used for remainder of the cycle. The same format was followed for cycle 3 of PEAQ-RT.

### Data analysis

The primary endpoint of this study was feasibility, defined as the successful completion of PEAQ-RT workflow in at least 80 % of attempted adaptive fractions. Radiation therapists recorded the average time for each step in the CTgART PEAQ-RT workflow, including setup, imaging, contour segmentation, plan re-optimization, treatment delivery, and plan quality assurance. Dosimetry data including dose to OARs, GTV, PTV, and projected dose from non-adaptive plans were also recorded prospectively.

## Results

### Patient characteristics

Patient demographics and disease characteristics are detailed in Table 1. A total of ten patients with age range of 56–89 years were accrued. All patients had primary head and neck malignancies of squamous cell histology, with their primary disease including oropharynx (4/10), larynx (3/10), hypopharynx (2/10), or oral cavity (1/10). QS-RT was indicated for all patients due to pain associated with bulky tumors. Half (5/10) of the patients had prior head and neck radiotherapy to a median dose of 7000 cGy (6600–7000 Gy). Median time from prior radiation to recurrence was 7 years (1–––20).

**Table 1:** Patient characteristics.

Patient #	Age	Primary site	Location of disease	Indication for Quad Shot	Presentation	Prior Therapy (RT, SysT, Sg)*	Number of Quad Shot cycles received	PFS (Months)	OS (Months)
1	63	Hypopharynx	Bilateral neck	Pain	Metastatic	None	2	0.43	0.86
2	67	Larynx	Right neck	Pain, tumor encroaching on tracheostomy	Localized	Sg, SysT, RT	3	4.0	4.0
3	63	Oropharynx	Oropharynx	Pain	Localized	Sg, SysT, RT	2	4.2	5.4
4	84	Oropharynx	Oropharynx	Pain, dysphagia	Localized	Sg, SysT, RT	3	1.1	1.1
5	56	Hypopharynx	Right neck	Pain, dysphagia	Localized	Sg	3	3.0	3.1
6	56	Larynx	Right neck	Pain	Localized	Sg, SysT, RT	3	2.6	2.6
7	62	Oropharynx	Right neck	Pain	Metastatic	SysT	2	2.1	2.1
8	84	Oral cavity	Right masticator space	Pain	Localized	Sg, SysT, RT	3**	1.0	3.0
9	73	Oropharynx	Right neck	Pain, dysphagia	Metastatic	None	1	0	0
10	89	Larynx	Larynx	Pain, odynophagia	Localized	None	1	0	0

* Sg-surgery, SysT-systemic therapy, RT-radiotherapy.

**PEAQ-RT workflow was not feasible for cycle 2 and 3 and patient treated with conventional QS-RT workflow.

### PEAQ-RT feasibility

**Fig. 2:**
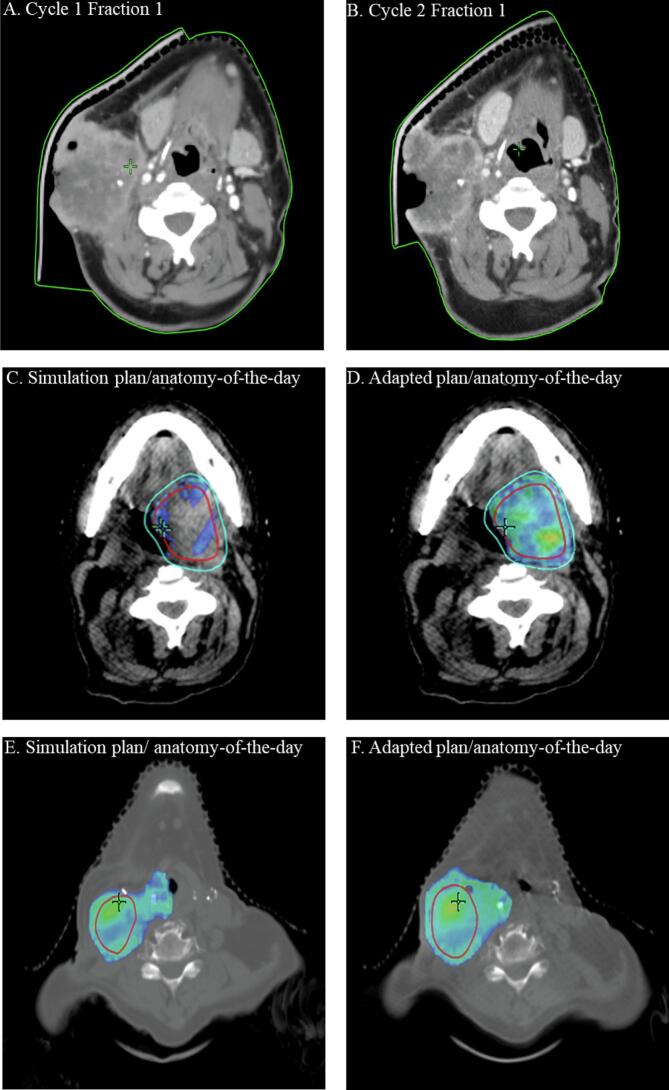
Benefits and limitations of PEAQ-RT online adaptive workflow. Panel A and B, demonstrated example of change in anatomy and immobilization device resulting in failed PEAQ-RT workflow. Panel C and D, 100% of prescrition dose to GTV/PTV coverage improved from scheduled plan to adapted plan from Cycle 1 to Cycle 2. Panel E and F, change in GTV size between Cycle 1 and Cycle 2, necessitating online adaptation.

Of the ten patients enrolled, all ten patients received their first cycle of PEAQ-RT. Eight of the ten patients received a second cycle of PEAQ-RT and six patients received a third cycle. The PEAQ-RT workflow was feasible in 87.5 % (7/8) of patients who received at least one adapted cycle and in 86 % (12/14) of all attempted adaptive fractions. All images acquired for ART were deemed adequate for contouring and re-optimization by the treating physician and physicist. Regarding the two infeasible fractions, one patient required repeat simulation for cycle 2 due to significant discrepancy between the deformed CT and CBCT images, prohibiting dose calculation. As shown in Fig. 2 A and B, this patient also required repeat simulation for cycle 3 due to an ill-fitting mask. For PEAQ-RT cycles 2 and 3, the average total workflow time for the first fraction adaptive treatment was 28 min (14–38).

### Dosimetric analysis of adaptation

For fraction 1 of cycle 2 and 3, online adaptation allowed for improved coverage of the GTV V100%Rx from 73.6 % (38.1 %-94.3 %) in the scheduled plan to 95.9 % (91.3 %-99.9 %) in the adaptive plan. PTV V95%Rx coverage improved from 89.3 % (81.1 %-99.2 %) in the scheduled plan to 97.3 % (89.4 %-99.9 %) in the adaptive plan. Example of improved GTV100%Rx coverage with PEAQ-RT is shown in Fig. 2 C and D. Median GTV volume changes from first cycle to second cycle were 11.08 cc (−35.26 cc-158.97 cc) and −14.69 cc (−114.42 cc-14.32 cc) from second cycle to third cycle. Example of GTV volume change requiring adaptation is shown in Fig. 2 E and F. Of the five patients with prior radiation, four finished all three cycles and one completed two cycles. Five out of the nine adaptive fractions the adaptive plan corrected an OAR dose violation (3 spinal cord, 2 brain).

### Treatment outcome

Eight out of ten patients (8/10) exhibited a reduction in size of their primary treated tumor after the first cycle. Two patients did not respond to treatment. Median overall survival (OS) was 3.75 months (0–9.5 months; 95 % CI: 0–7.6 months), and the median progression-free survival (PFS) was 2.0 months (0–8.23 months; 95 % CI: 0–5.1 months). One patient experienced Grade 3 soft tissue necrosis of the neck mass after the first cycle of QS-RT. No other acute treatment related toxicities were reported.

### Treatments burden

PEAQ-RT workflow eliminated 12 extra simulations total for enrolled patients (Table 2). This resulted in a total of 1,200.6 travel miles saved by the patients, with a median of 50.8 (40.6–848) miles saved per patient. Assuming patients traveled by passenger car with a fuel efficiency of 25 MPG, this equates to a total of 426.71 kg of CO2 emissions saved. When considering both simulations and travel time, PEAQ-RT saved patients a total of 33 h, with a median of 3.5 h per patient (2–14.6 h).

**Table 2:** Summary statistics of patient travel and time savings.

	# of Simulations Eliminated	Distance From Treatment Center	Travel Distance Saved	Travel Time Saved	Simulation Time Saved	All Time Saved	CO2 Emissions Saved
Median	2	24.1 miles	59 miles	1.5 h	2 h	3.5 h	21 kg
Mean	1.2	52.6 miles	171.5 miles	3 h	1.7 h	4.7 h	61 kg
Range	0–2	1.9–––212 miles	40.6–––848 miles	1–12.6 h	1–––2 h	2–14.6 h	14.4–301 kg
Total	12	526.2 miles	1200.6 miles	21 h	12 h	33 h	427 kg

## Discussion

QS-RT is an effective form of palliative radiotherapy for patients with head and neck cancers [1,2]. Emerging data suggest that QS-RT combined with immunotherapy may have a synergistic mechanism that improves disease control for patients with incurable head and neck malignancies [11]. Despite this, only 7 % of patients with head and neck malignancies receiving palliative treatment receive QS-RT, suggesting that this regimen is underutilized [12]. While reasons for this underutilization are likely multi-factorial, the need for repeat CT simulations compared to a single CT simulation appointment for conventional palliative radiotherapy is a workflow-defined barrier to more frequent utilization.

The PEAQ-RT workflow highlights several advantages of combining QS-RT with CTgART. CTgART eliminated the need for CT simulation appointments for additional QS-RT cycles. Additionally, PEAQ-RT demonstrated dosimetric benefits, improving target coverage and resolving OAR constraint violations when comparing simulation plans to online adaptive plans. This is particularly critical in this population as many patients receiving QS-RT have previously undergone radiotherapy.

A significant proportion of patients at our institution reside in rural areas with limited access to radiation oncology services, with 40 % living more than 30 miles away and 25 % living more than 60 miles away [17]. Patients with limited geographic access to radiation treatment centers have poorer outcomes, making it crucial to optimize treatment planning and logistical arrangements for this vulnerable patient population [13,14],[15],[16]. The PEAQ-RT workflow demonstrated tangible benefits by reducing the number of trips required for simulations, as well as the miles traveled and hours spent on these additional appointments. These reductions translate into direct cost savings, such as decreased fuel expenses, and indirect benefits, including reduced caregiver burden. Ultimately, these improvements can have a meaningful impact on patients' quality of life.

Several considerations for our trial should be noted. Implementing online adaptation demands a substantial investment of planning and machine time, consuming valuable personnel, and physician resources. Therefore careful selection of patients who will most benefit from PEAQ-RT is essential, especially in centers with high adaptive volumes. Relatedly, onboard CBCT imaging must be of sufficient quality to enable accurate target and OAR delineation for adaptive replanning and dose calculation. However, advancements in onboard imaging modalities may mitigate this limitation by providing diagnostic-quality imaging for treatment planning. Additionally, PEAQ-RT includes scheduling a simulation slot in case online adaptation proves unsuccessful, potentially occupying a time slot for simulation that could have been allocated to other patients. Finally, some patients did have trouble laying on treatment table with immobilization mask for the duration of PEAQ-RT workflow, though all patients were able to complete the attempted fractions.

This pilot study shows that expedited QS-RT using PEAQ-RT is feasible and reduces patient burden by eliminating the need for CT simulation. While technically involved, PEAQ-RT offers meaningful benefit for select patients, particularly those with significant travel barriers, making it a valuable option for centers with adaptive capabilities.

## AI statement

During the preparation of this work the author(s) used ChatGPT in order to correct spelling errors, typos and grammar mistakes. After using this tool/service, the author(s) reviewed and edited the content as needed and take(s) full responsibility for the content of the publication.

## CRediT authorship contribution statement

**Weiren Liu:** Writing – original draft, Writing – review & editing, Investigation. **Joshua P. Schiff:** Writing – review & editing, Investigation, Visualization, Supervision. **Comron Hassanzadeh:** Conceptualization, Methodology. **Karen Miller:** Resources. **Casey Hatscher:** Resources. **Robbie Beckert:** Resources. **Alex Price:** Conceptualization, Methodology. **Mackenzie Daly:** Writing – review & editing. **Randall Brenneman:** Writing – review & editing. **Lauren Henke:** Conceptualization, Methodology. **Anthony Apicelli:** Writing – review & editing. **Michael Moravan:** Writing – review & editing. **Wade Thorstad:** Conceptualization, Supervision, Writing – review & editing. **Eric Laugeman:** Formal analysis, Data curation, Conceptualization, Supervision, Writing – review & editing.

## Funding

This study was funded by Varian Medical Systems. The sponsor had no role in the study design, in the collection, analysis, and interpretation of the data, in the writing of the report, and in the decision to submit the article for publication.

## Declaration of competing interest

The authors declare the following financial interests/personal relationships which may be considered as potential competing interests: Dr. Wade Thorstad received funding paid to the institution in support of this clinical trial. Washington University receives research support from Varian Medical Systems, Siemens, Mevion, and ViewRay unrelated to the present work. All other conflicts of interest are unrelated to the present work.

## Data Availability

Research data are stored in an institutional repository and will be shared upon request to the corresponding author.
